# Efficacy and Potential Mechanisms of Umbilical Cord‐Derived Mesenchymal Stem Cells in the Treatment of Ischemic Stroke in Animal Models: A Meta‐Analysis

**DOI:** 10.1111/cns.70357

**Published:** 2025-04-09

**Authors:** Renli Wei, Minguang Yang, Yue Cao, Shuqian Qiu, Xu Fan, Muxuan Fang, Li Chen, Shaojie Cheng, Jianhong Li, Shenghang Zhang

**Affiliations:** ^1^ Fujian Key Laboratory of Aptamers Technology Fuzhou General Teaching Hospital (The 900th Hospital), Fujian University of Traditional Chinese Medicine Fuzhou Fujian China; ^2^ College of Rehabilitation Medicine Fujian University of Traditional Chinese Medicine Fuzhou Fujian China; ^3^ The Institute of Rehabilitation Industry Fujian University of Traditional Chinese Medicine Fuzhou Fujian China; ^4^ Department of Clinical Laboratory Medicine Fuzhou General Clinical Medical School, Fujian Medical University Fuzhou Fujian China; ^5^ Fujian Key Laboratory of Aptamers Technology 900th Hospital of Joint Logistics Support Force, People's Liberation Army (PLA) Fuzhou Fujian China

**Keywords:** animal study, ischemic stroke, meta‐analysis, umbilical cord‐derived mesenchymal stem cells

## Abstract

**Background:**

Umbilical cord‐derived mesenchymal stem cells (UCMSCs) have emerged as a promising treatment for ischemic stroke. This study aimed to evaluate the therapeutic efficacy and potential mechanisms of UCMSCs in treating ischemic stroke.

**Methods:**

A systematic search of PubMed, Web of Science, and Embase was conducted up to April 25, 2024. Literature was screened based on the PICOS principle, with predefined inclusion and exclusion criteria. Relevant data were extracted and analyzed using Review Manager 5.4.

**Results:**

Out of 1390 retrieved articles, 30 were included in the meta‐analysis. UCMSCs significantly reduced infarct size and volume, improved neurological deficit scores, and facilitated neurobehavioral recovery. UCMSCs treatment also modulated inflammatory cytokine levels in brain tissue and serum, promoted microglial polarization, inhibited apoptosis, and increased vessel density in the peri‐infarct tissue.

**Conclusions:**

UCMSCs administration significantly promoted the neurological function recovery after ischemic stroke. Their mechanisms of action may be related to immune response regulation, inhibition of apoptosis, and promotion of angiogenesis. These findings provide theoretical guidance for improving the quality of basic research and clinical translation.

## Graphical abstract

Efficacy and potential mechanisms of UCMSCs in IS animals.

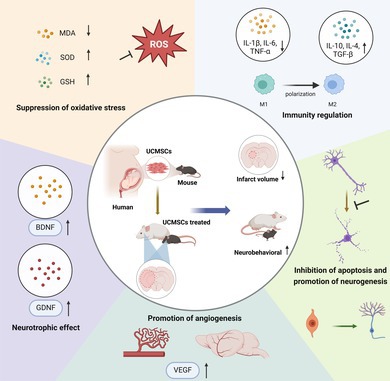

## 1 Introduction

Ischemic stroke (IS) is the most common stroke type, which accounts for 60%–70% of all strokes [1]. Ischemic stroke is characterized by insufficient blood supply to the brain, triggering a series of pathophysiological responses, including alterations in energy metabolism, neuroinflammation, oxidative stress, and neuronal apoptosis, which ultimately result in severe neurological impairment [2, 3]. As a cerebrovascular disease, IS seriously affects patients' quality of life and causes a heavy social and economic burden [4, 5]. Recombinant tissue plasminogen activator and endovascular thrombectomy are commonly employed for acute IS in clinical practice. However, due to the limited therapeutic window, a significant number of patients do not receive timely and efficacious treatment, often resulting in irreversible nerve damage [6, 7]. The limited effectiveness of current treatment options for IS necessitates an urgent need for new therapies that can promote the recovery of neurological function following IS.

In recent years, mesenchymal stem cells (MSCs) therapy has emerged as a promising treatment for central nervous system injuries, gradually becoming a research focus [8, 9]. MSCs transplantation has been shown to inhibit neuronal apoptosis, promote neurogenesis, expedite angiogenesis, and regulate immunity [8]. Among various MSC sources, umbilical cord‐derived mesenchymal stem cells (UCMSCs) have significant advantages because of their ease of acquisition, non‐invasiveness, lack of harm to the donor, and ethical compliance [10]. UCMSCs are also characterized by low carcinogenicity, pluripotency, high immune regulatory capacity, and low immunogenicity [11].

UCMSCs have attracted considerable attention for their therapeutic potential in the treatment of IS. Preclinical animal studies have demonstrated significant benefits in reducing infarct volume and promoting neurological recovery after IS [12, 13, 14, 15]. Additionally, previous clinical studies have shown that stroke patients treated with UCMSCs can significantly improve spasticity and motor function, leading to enhanced quality of life without any reported adverse events [16].

A previous meta‐analysis study of MSCs showed that MSC therapy exhibited favorable therapeutic effects on a variety of neurological functions and motor abilities in animal models of subacute or chronic stroke [17]. Herein, we performed a meta‐analysis to assess the effects of UCMSCs in animal models of IS. Three databases (PubMed, Web of Science, Embase) were systematically searched, and articles involving the application of UCMSCs in IS animal models were included for analysis. The aim of this study was to evaluate the therapeutic effects and potential mechanisms of UCMSCs on brain injury and neurological function in ischemic stroke.

## 2 Methods

### 2.1 Data Sources and Search Strategy

The meta‐analysis was conducted in accordance with the Preferred Reporting Items for Systematic Reviews and Meta‐Analyses (PRISMA). Relevant literature related to “umbilical cord mesenchymal stem cells” and “ischemic stroke” was retrieved from the PubMed, Web of Science, and Embase databases, with databases from inception until April 25, 2024. The search strategy consisted of the following terms: “umbilical cord mesenchymal stem cells”, “mesenchymal stem cells”, “umbilical cord mesenchymal stromal cells”, “UCMSCs”, “Wharton's Jelly”, or “WJ‐MSCs” and “ischemic stroke”, “brain ischemia”, “cerebral infarct”, “cerebral ischemic/reperfusion”, “middle cerebral artery occlusion”, or “MCAO”. To ensure a comprehensive coverage, a thorough search of the references in relevant literature was performed to avoid the omission of pertinent research. This review has been registered in the International Registry of Prospective Systematic Reviews PROSPERO under the registration number: CRD42024529748.

### 2.2 Inclusion and Exclusion Criteria

The inclusion criteria were as follows: (1) utilization of an animal model for IS research; (2) intervention with UCMSCs; (3) inclusion of a corresponding control group; and (4) studies published in English with full text availability.

The exclusion criteria were as follows: (1) duplicate documents; (2) reviews, conference abstracts, comments, etc.; (3) clinical trials and in vitro studies; and (4) studies with no relevant, extractable results.

### 2.3 Study Selection

The studies were imported into NoteExpress for the removal of any duplicate documents. An initial examination of the titles and abstracts of relevant articles was conducted independently by two investigators. Full‐text articles were then independently reviewed according to predetermined inclusion and exclusion criteria to determine which studies would be included. Any disagreements were resolved through a third investigator.

### 2.4 Data Extraction

The data from eligible studies were extracted independently by two investigators, and any discrepancies were resolved through discussion with a third investigator. Information regarding the study authors, publication year, author country, stroke model, animal types, dose of UCMSCs, time of administration, route of administration, and outcome assessments was collected. For studies in which the specific numerical results were not presented, data were extracted from the graphs by GetData Graph Digitizer software (version 2.24). Means and standard deviations (SDs) were extracted, and in cases where SDs were not reported, standard errors were converted to SDs by using the calculator function of Review Manager 5.4 software.

When results at multiple time points were available, the outcome measure at the final time point was extracted. Additionally, if data from more than one brain tissue region were reported, only the cortical region on the side of the ischemic lesion was extracted. If a study involved multiple doses, time windows, or routes of UCMSCs transplantation, the data were extracted and analyzed as a whole.

### 2.5 Quality Assessment

The quality of the included studies was independently assessed by two investigators using the Systematic Review Centre for Laboratory Animal Experimentation (SYRCLE) bias risk tool [18]. Ten items were used to evaluate the risk of bias in the animal studies, including random sequence generation, similarity of groups baseline, allocation concealment, random housing of animals, blinding of caregivers and/or examiners, random selection for outcome assessment, blinding of outcome assessors, addressing incomplete outcome data, selective outcome reporting, and other potential sources of bias. Any discrepancies were resolved through discussion with a third investigator.

### 2.6 Statistical Analysis

Data aggregation was conducted using Review Manager 5.4. The combined effect size was calculated as a standardized mean difference (SMD) and a 95% confidence interval (CI) between the UCMSCs treatment and control groups, and forest plots were generated. All statistical tests were performed two‐sided, and differences were considered significant when *p* < 0.05. Heterogeneity was assessed using *I* ^2^, with *I* ^2^ ≤ 50% indicating low heterogeneity, where a fixed effects model was employed, while *I* ^2^ > 50% indicated high heterogeneity, requiring the use of a random effects model.

## 3 Results

### 3.1 Study Selection Process

**FIGURE 1:**
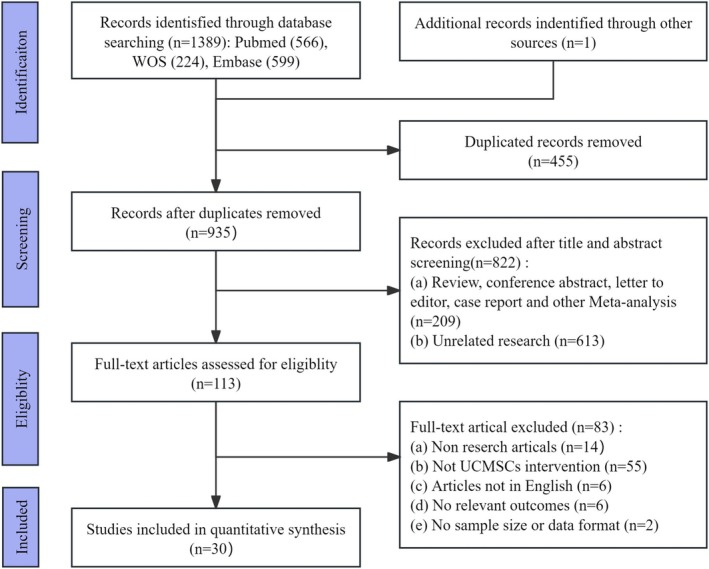
PRISMA flow diagram.

A total of 1390 potentially relevant articles were identified through the literature search, which included 566 from PubMed, 224 from Web of Science, 599 from Embase, and 1 from the references of the included articles. After the exclusion of 455 duplicate publications, 935 articles were available for further screening. Based on the evaluation of titles and abstracts, 822 articles were excluded. A full‐text review was then performed on the remaining 113 articles. In accordance with the inclusion and exclusion criteria, 83 articles were further excluded: 14 were non‐experimental articles, 55 did not involve UCMSCs treatment, 6 were not available in full English, 6 lacked relevant outcome measures, and 2 failed to provide sample size or data form. Ultimately, 30 articles were considered suitable for inclusion in this study. The detailed flow of literature screening is illustrated in Figure 1.

### 3.2 Study Characteristics

The characteristics of the included studies are as follows Table 1. The included studies were published between 2007 and 2024 and originated from various countries, including China, Korea, the United States, Italy, Germany, and Japan. Rats were used in 24 studies, while 6 studies employed mice as animal models. UCMSCs from human sources were used in all 30 studies, and one study also included UCMSCs derived from mice [25]. Regarding the injection methods, 16 studies administered UCMSCs intravenously, 11 employed the intracerebral route, 1 study used the arterial route, 1 compared intracerebral and intravenous routes, and 1 combined arterial and intravenous injections. In terms of dosing regimens, 16 studies administered UCMSCs at doses of ≥ 1 × 10^6^ cells, while 7 studies used doses of < 1 × 10^6^ cells. An additional 7 studies explored various UCMSCs dosing regimens.

**TABLE 1:** Characteristics of the included studies.

Author	Country	Animals	Model	Types of UCMSC	Source of UCMSC	Time of administration	Route of administration	Control	Dose	Frequency	Follow‐up	Outcomes
Cao, 2020 [19]	China	Adult Sprague Dawley rats weighting 250–300 g	MCAO	HUCMSCs	Obtained from the Stem Cell Bank	24 h	IC	PBS	1 × 10^6^ cells in 50 mL PBS	1	7 days after MCAO	Percentage of cerebral infarction area, mNSS
Cheng, 2015 [13]	China	6–7‐week‐old male mice	MCAO	HUCMSCs	Umbilical cord	30 min	IV	Normal saline	4 × 10^6^ cells in 0.5 mL normal saline	1	24 h, 72 h, 1 week after MCAO	Percentage of cerebral infarction volume, brain water content, neurological score
Choi, 2016 [20]	Korea	Male Sprague–Dawley rats	MCAO	HUCMSCs	Umbilical cord of a single healthy donor	2 h	IV	Normal saline	1 × 10^6^ cells mixed with 500 μL saline	1	4 weeks after MCAO	Percentage of cerebral infarction area, mNSS, Iba‐1 positive cells
Choi, 2018 [21]	Korea	Eight‐week‐old rats Male Sprague–Dawley rats weighing 270–300 g	MCAO	HUCMSCs	The umbilical cord from healthy donor	29 days, 31 days	IV	PBS	1× 10^6^ cells in 0.5 mL PBS	2	28 days after UCMSCs treatment	Proliferation of neuroblasts, BrdU‐labeled cells proliferating, mNSS
Ding, 2007 [22]	China	Adult male Sprague–Dawley rats weighting 250–300 g	MCAO, CCAO	HUCMSCs	Human umbilical cord samples	1 week	IC	PBS	1 × 10^6^ cells in a 3–5 μL PBS	1	35 days after MCAO	Body asymmetry test, locomotor activity, vessel density
Fu, 2022 [12]	China	Male Sprague–Dawley rats weighing 280–320 g	MCAO, CCAO	HUCMSCs	The mesenchymal tissue in Wharton's jelly	14 days	IV	Normal saline	1 × 10^6^ cells in PBS	1	56 days after MCAO	Retention time, cylinder test, percentage of vessel density
Karlupia, 2014 [23]	USA	Adult male Wistar rats	MCAO	HUCMSCs	Obtained commercially from PromoCell	24 h	IC	PBS	5 × 10^6^ cells in PBS	1	14 days after MCAO	Percentage of cerebral infarction area, rotarod test
Koh, 2008 [24]	Korea	Sprague–Dawley rats weighing 295 to 360 g	MCAO	HUCMSCs	Human umbilical cord	2 weeks	IC	PBS	6 × 10^5^ cells in 5 μL PBS	1	35 days after MCAO	Infarct volume size, neurological deficit score
Li, 2013 [25]	China	Wild‐type 1‐week‐old male c57bl/6jnarl mice	MCAO, CCAO	HUCMSCs, mUCMSCs	① HUCMSCs were kindly provided gratis from Dr. Woei‐Cherng Shyu ② Mouse umbilical cords were aseptically collected from C57BL/6JNarl	24 h	IC	PBS	1.6 × 10^6^ cells	1	14 days after MCAO	Infarct volume size
Li, 2015 [26]	China	54 Sprague–Dawley pathogen‐free rats of both sexes	MCAO	HUCMSCs	Human umbilical cords were obtained from full‐term deliveries	Reperfusion	IV	PBS	3 × 10^6^ cells in 600 μL saline	1	24 h and 14 days after MCAO	Percentage of cerebral infarction volume, BrdU labeled cells proliferating
Li, 2023 [27]	China	Adult male C57BL/6 mice weighing 22–25 g (6–8 weeks)	MCAO	HUCMSCs	Clinical‐grade HUCMSCs	30 min	IV	Normal saline	0.2 mL saline at 10^6^ cells/20 g mouse	1	21 days after MCAO	Infarct volume size, brain water content, IL‐1β, IL‐6, TNF‐α, IL‐4, IL‐10, TGF‐β in brain tissue
Liao, 2009 [28]	China	Adult male Sprague–Dawley rats	MCAO	HUCMSCs	Both sexes were collected from full‐term caesarian section deliveries	24 h	IC	PBS	2 × 10^5^ cells in 10 μL PBS	1	5 weeks after MCAO	Percentage of cerebral infarction volume, mNSS, Morris water maze test, percentage of vessel density
Lin, 2011 [14]	China	Adult Sprague–Dawley rats weighing 280–360 g	MCAO	HUCMSCs	Human umbilical mesenchymal stem cells in the Wharton's jelly of the umbilical cord	24 h	IC	PBS	5 × 10^5^ cells	1	36 days after transplantation	Infarct volume size, retention time, cylinder test, percentage of vessel density
Lin, 2017 [29]	China	Adult male, Sprague–Dawley rats weighing 300–350 g	MCAO	HUC‐MSCs	Umbilical cord tissue	24 h	IV	Normal saline	1 × 10^6^, 4 × 10^6^ cells/mL/kg of body weight	1	14 days after MCAO	Infarct volume size， mNSS, adhesive removal test, Iba‐1 positive cells, apoptosis rate, proliferation of neuroblasts
Lin, 2023 [30]	China	Adult male Sprague–Dawley rats weighting 200–250 g	MCAO, CCAO	HUCMSCs	Human umbilical cord tissues Wharton's jelly	30 min, 24 h	IV, IA	Normal saline	3 × 10^6^, and 1 × 10^5^ cells 500 μL saline	2	28 days after MCAO	Infarct volume size, apoptosis rate
Liu, 2010 [31]	China	Adult male Sprague–Dawley rats	MCAO， CCAO	HUCMSCs	Human umbilical cord samples	1 week	IC	PBS	1 × 10^6^ cells in a 3‐5 μL PBS	1	36 days after transplantation	Body asymmetry test, locomotor activity
Noh, 2020 [32]	Korea	Adult male Sprague–Dawley rats weighing 270–300 g	MCAO	HUCMSCs	Human umbilical cord samples	1 week	IV, IC	Normal saline	1 × 10^6^ cells in 500 μL of saline or 1 × 10^6^ cells in 8 μL of saline	1	8 weeks after MCAO	Percentage of cerebral infarction volume, mNSS, rotarod test, Iba‐1 positive cells, ED1^+^ (%), proportion of iNOS^+^/ED1^+^ cells, proportion of CD206^+^/ED1^+^ cells
Oh, 2018 [33]	Korea	Male Sprague–Dawley rats weighing 270–300 g	MCAO	HUCMSCs	The umbilical cord from healthy donor	24 h, 7 days	IV	Normal saline	1 × 10^5^ cells, 5 × 10^5^ cells, 1 × 10^6^ cells in 500 μL of saline	1	8 weeks or 4 weeks after MCAO	Percentage of cerebral infarction area, mNSS, rotarod test, Iba‐1 positive cells, ED1 + (%), proportion of iNOS+/ED1+ and CD206^+^/ED1^+^ cells, apoptosis rate
Sabbaghziarani, 2017 [34]	Germany	Male Wistar albino rats Weighing 270–300 g (12‐week‐old)	MCAO	HWJ‐MSCs	Umbilical cords were obtained with the signed permission of the Arash Hospital patients	24 h	IV	NR	1 × 10^6^ cells	1	7 days after MCAO	TNF‐α, IL‐6 in serum
Shams, 2015 [35]	Iran	Male Sprague–Dawley rats with a body weighing 240–280 g	MCAO	HUCMSCs	Umbilical cords were obtained from healthy mothers delivering full‐term infants by cesarean section	24 h	IV	PBS	1 × 10^6^ cells in 50 mL PBS	1	16 days after MCAO	Infarct volumes size, Morris water maze test
Shen, 2022 [15]	China	Rats	MCAO	HUCMSCs	The umbilical cords of newborns	First on Day 1, 4, 7, and 14 after MCAO, and twice was given 7 days after the first administration	IC	PBS	1 × 10^7^ cells/kg, 5 × 10^6^ cells/kg, 2 × 10^7^ cells/kg	2	14 days after MCAO or 28 days after the first administration	Percentage of cerebral infarction area; IL‐10, IL‐6, TNF‐α, IL‐1β in serum and brain tissue
Sun, 2020 [36]	China	Adult Sprague Dawley rats weighing 250–300 g	MCAO, CCAO	HUCMSCs	Human umbilical cord tissues Wharton's jelly	60 min	IC	PBS	1 × 10^5^ cells in 3 μL of HUCMSC complete medium	1	28 days after MCAO	IL‐6, IL‐10, IL‐1β in brain tissue; apoptosis
Tanaka, 2018 [37]	Japan	Cb17 male and female mouse pups 12 days postpartum	MCAO	HUCMSCs	Women who underwent cesarean sections	48 h	IV	Cryoprotectant	1 × 10^4^, 1 × 10^5^ cells in 60 μL of STEM‐CELLBANKER cryoprotectant	1	Postnatal Day 28	Rotarod test, cylinder test, Iba‐1 positive cells
Teng, 2021 [38]	China	Adult male Sprague–Dawley rats weighting of 320–350 g	MCAO	HUCMSCs	Umbilical cord tissue	24 h	IV	Normal saline containing 2% clinical grade HSA and 16.7% clinical grade CS10	3.3 × 10^5^, 1 × 10^6^, or 1 × 10^6^ cells	1	14 days after MCAO	Percentage of M1 and M2 cells
Wu, 2018 [39]	China	Adult Sprague–Dawley rats weighing 270–300 g	MCAO	HUCMSCs	Umbilical cord tissue	24 h	IV	Normal saline	6.67 × 10^4^ viable cells/μL × 5 μL culture media	1	5 days after MCAO	Infarct volumes size, body asymmetry test, Iba‐1 positive cells
Yang, 2024 [40]	China	Male C57BL/6 mice weighing approximately 20 g	MCAO	HUCMSCs	Human umbilical cord	24 h	IV	Normal saline	5 × 10^5^ cells	1	28 days after MCAO	Percentage of cerebral infarction area, IL‐1β, IL‐6, TNF‐α, IL‐4, IL‐10, TGF‐β in brain tissue，Iba‐1 positive cells
Zhang, 2011 [41]	USA	Male Wistar rats weighing 270 to 300 g	MCAO	HUTC	Human umbilical tissue	24 h	IV	PBS	3 × 10^5^, 1 × 10^6^, 3 × 10^6^, 1 × 10^7^, or 5 × 10^6^ cells in 2 mL PBS	1	60 day, 140 day after MCAO	Percentage of cerebral infarction volume, mNSS, adhesive removal test, apoptotic cells, proliferation of neuroblasts, BrdU‐labeled cells proliferating, vessel density
Zhang, 2017 [42]	China	Adult female specific‐pathogen‐free Sprague–Dawley	MCAO	HWJ‐MSCs	Umbilical cord tissue	2 days	IV	Normal saline	1 × 10^7^ cells in 200 μL cells	1	35 days after HWJ‐MSCs transplantation	Longa scoring, rotarod test, Morris water maze test
Zhu, 2015 [43]	China	Adult male C57BL6/J mice 8–10 weeks age	MCAO	HUCMSCs	Umbilical cord of informed, healthy mothers	24 h	IC	PBS	1.5 × 10^5^ cells in PBS	1	14 day and 28 day after MCAO	Percentage of cerebral infarction volume, mNSS, retention time, adhesive removal test, vessel density
Zuo, 2019 [44]	China	7–8 weeks old specific pathogen‐free Sprague–Dawley rats weighing 240–280 g	MCAO	HUCMSCs	Provided by Prof. Yufang Shi and his team	24 h	IV	Normal saline	2 × 10^6^ cells in 0.5 mL normal saline	1	2, 3 and 7 days after MCAO	Percentage of cerebral infarction volume, apoptosis rate

Abbreviations: CCAO, common carotid artery occlusion; HSA, human serum albumin; HUCMSCs, human umbilical cord‐derived mesenchymal stem cells; HUTC, Human umbilical cord tissue‐derived cells; HWJ‐MSC, human Wharton's jelly mesenchymal stromal cell; IC, intracerebral injection; IL‐1β, interleukin‐1β; IL‐10, interleukin‐10; IL‐4, interleukin‐4; IL‐6, interleukin‐6; IV, intravenous; MCAO, middle cerebral artery was occluded; mNSS, modified Neurological Severity Score; mUCMSCs, mouse derived umbilical cord mesenchymal stem cells; PBS, phosphate buffer saline; TGF‐β, transforming growth factor‐beta; TNF‐α, tumor necrosis factor‐α.

### 3.3 Study Quality

**FIGURE 2:**
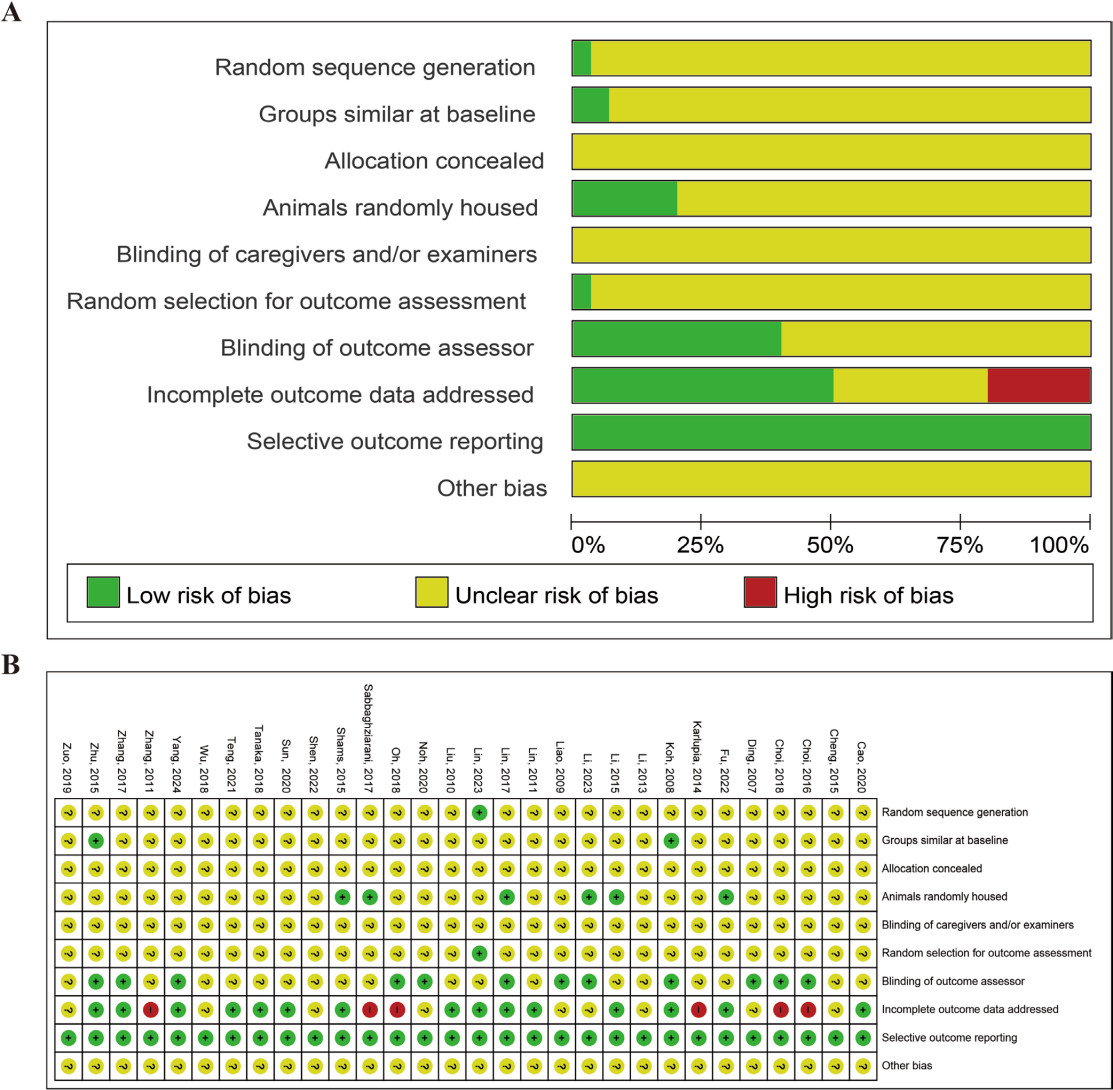
SYRCLE risk of bias assessment for included studies. (A) Risk of bias graph. (B) Risk of bias summary.

The risk of bias for the included studies was evaluated using the Systematic Review Centre for Laboratory Animal Experimentation (SYRCLE) bias risk tool, with the summarized results presented in Figure 2. Among the 30 included studies, only one study explicitly described the use of a table of random numbers for experimental group allocation, while others indicated randomization without providing specific details, resulting in an unclear risk of bias. Baseline characteristics such as age, weight, and sex of the animals were reported by most studies; however, the influence of additional factors could not be excluded and was assessed as uncertain. None of the studies adequately described the methods used for allocation concealment. Additionally, blinding of caregivers or examiners was not explicitly mentioned in any study, although some indicated that outcome assessors were blinded. Randomization in outcome assessment was only reported in one study. Due to the limited available information, it remains unclear whether bias from other sources could have been present. Overall, the included studies displayed unclear risks across many criteria, though the overall risk of bias was not considered high.

### 3.4 UCMSCs Treatment Alleviated Cerebral Infarction in IS Animals

**FIGURE 3:**
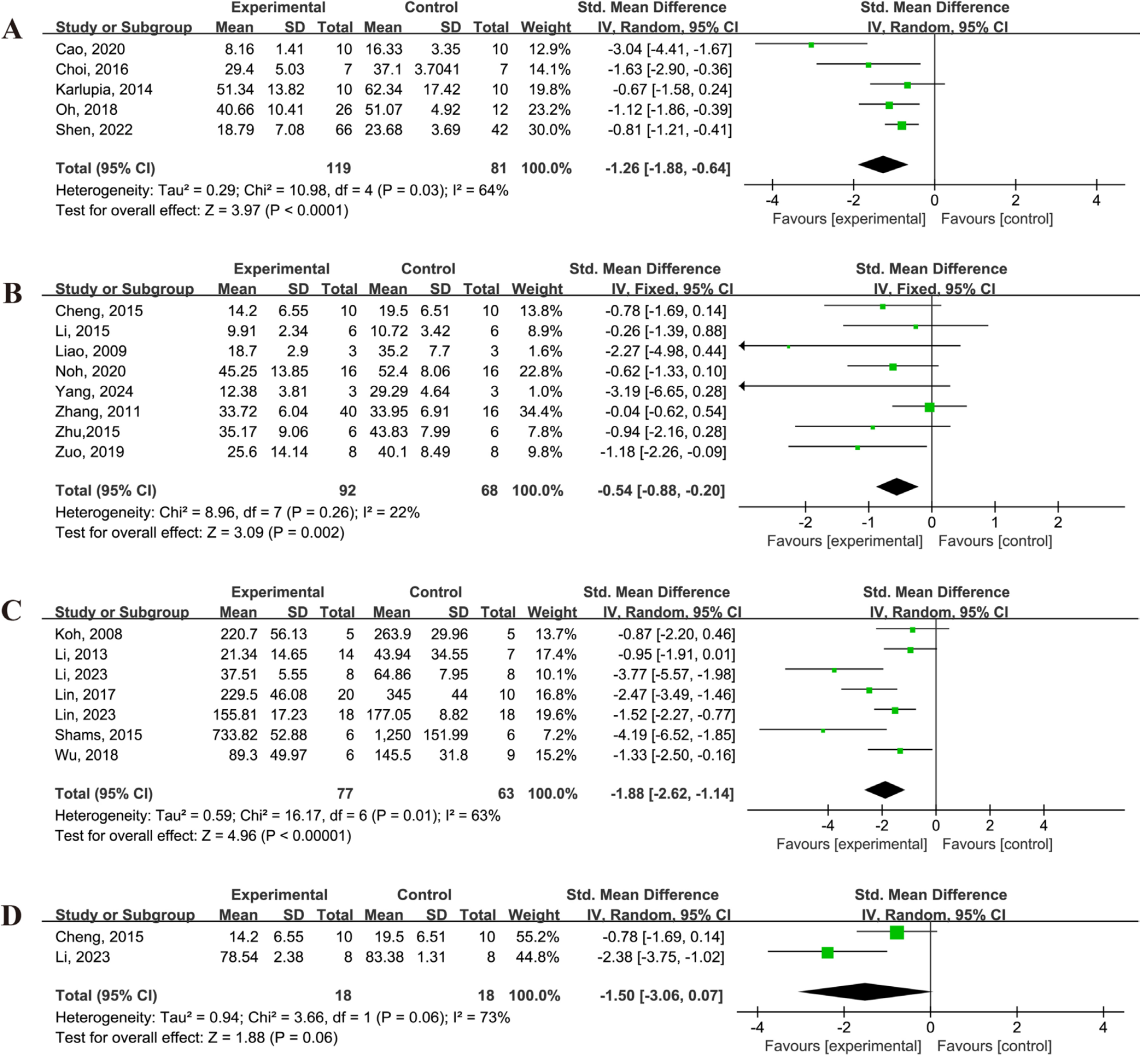
Forest plot demonstrating the efficacy of UCMSCs on cerebral infarct size and edema in IS animals. (A) Percentage of cerebral infarction area. (B) Percentage of cerebral infarction volume. (C) Infarct volume size. (D) Brain water content.

A total of 20 studies reported changes in infarct size following UCMSCs treatment in IS animals, as assessed by TTC staining or MRI (Figure 3). Of these, five studies [15, 19, 20, 23, 33] reported the percentage of infarct area, eight studies [13, 26, 28, 32, 40, 41, 43, 44] reported the percentage of infarct volume, and seven studies [24, 25, 27, 29, 30, 35, 39] reported infarct volume size. UCMSCs treatment was found to significantly reduce the percentage of infarct size (SMD = −1.26, 95% CI: −1.88 to −0.64, *I* ^2^ = 64%), the percentage of infarct volume (SMD = −0.54, 95% CI: −0.88 to −0.20, *I* ^2^ = 22%) and infarct volume size (SMD = −1.88, 95% CI: −2.62 to −1.14, *I* ^2^ = 63%) (Figure 3A–C). Additionally, 2 studies [13, 27] assessed brain water content after UCMSCs treatment; however, no significant reduction in brain edema was observed (SMD = −1.50, 95% CI: −3.06 to 0.07, *I* ^2^ = 73%) (Figure 3D).

### 3.5 UCMSCs Treatment Improved the Neurological Deficit Score in IS Animals

**FIGURE 4:**
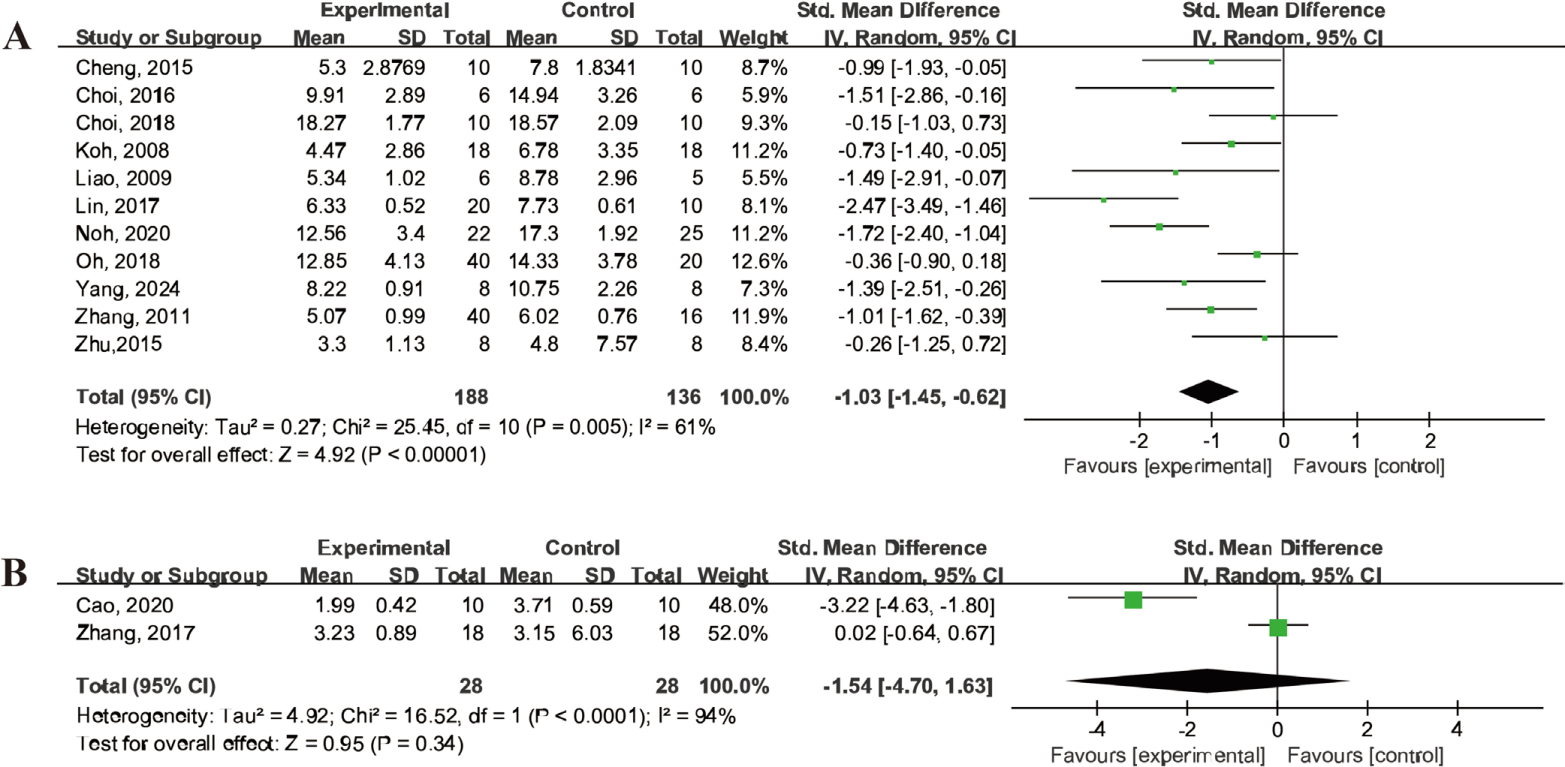
Forest plot illustrating the efficacy of UCMSCs on neurological scores in IS animals. (A) mNSS. (B) Longa.

A total of 11 studies [13, 20, 21, 24, 28, 29, 32, 33, 40, 41, 43] reported improvements in the modified Neurological Severity Score (mNSS), with significant enhancements observed following UCMSCs treatment (SMD = −1.03, 95% CI: −1.45 to −0.62, *I* ^2^ = 61%) (Figure 4A). However, two studies [19, 42] reported Longa scores 3 days after MCAO, and the results showed that UCMSCs treatment did not significantly reduce Longa scores (SMD = −1.54, 95% CI: −4.7 to 1.63, *I* ^2^ = 94%) (Figure 4B).

### 3.6 UCMSCs Treatment Promoted Neurobehavioral Recovery in IS Animals

**FIGURE 5:**
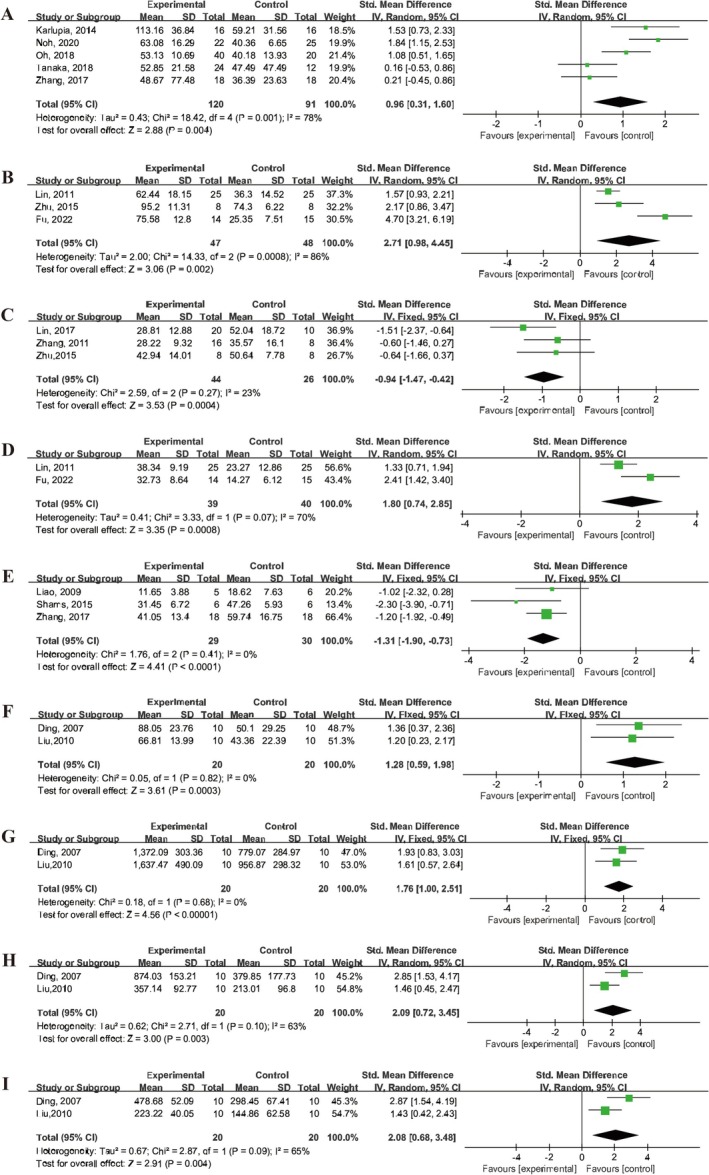
Forest plot depicting the efficacy of UCMSCs on neurological behaviors in IS animals. (A) Rotarod test. (B) Retention time. (C) Adhesive removal test. (D) Cylinder test. (E) Morris water maze test. (F) Body asymmetry test. (G) Vertical activity. (H) Vertical movement time. (I) Number of vertical movements.

Several behavioral tests were employed to assess neurological function, including the rotarod test, adhesive removal test, cylinder test, Morris water maze test, body asymmetry test, and various locomotor activity parameters (vertical activity, vertical movement time, and the number of vertical movements) (Figure 5).

In the rotarod test, five studies [23, 32, 33, 37, 42] reported the time spent by animals on the rotarod, while three studies [12, 14, 43] reported the percentage of time spent on the rotarod compared to baseline. The meta‐analysis demonstrated a significant increase in the time on the rotarod in animals treated with UCMSCs (SMD = 0.96, 95% CI: 0.31 to 1.6, *I* ^2^ = 78%) and retention time (SMD = 2.71, 95% CI: 0.98 to 4.45, *I* ^2^ = 86%) (Figure 5A,B). In addition, three studies [29, 41, 43] assessed the adhesive removal test, showing a reduction in adhesive removal time in IS animals treated with UCMSCs (SMD = −0.94, 95% CI: 1.47 to 0.42, *I* ^2^ = 23%) (Figure 5C). The cylinder test, used in two studies [12, 14], revealed that UCMSCs treatment improved the asymmetry of forelimb use in IS animals (SMD = 1.8, 95% CI: 0.74, 2.85, *I* ^2^ = 70%) (Figure 5D).

Cognitive function, as assessed by the Morris water maze test in three studies [28, 35, 42], reported that UCMSCs treatment reduced the escape latency time in IS animals (SMD = −1.31, 95% CI: −1.90 to −0.73, *I* ^2^ = 0%) (Figure 5E).

The body asymmetry test, reported in two studies [22, 31], indicated that UCMSCs treatment promoted the recovery of body symmetry in IS animals (SMD = 1.28, 95% CI: 0.59 to 1.98, *I* ^2^ = 0%) (Figure 5F). Additionally, one study [39] reported a significant reduction in rotation frequency of the upper limb or head towards the ipsilateral side in UCMSCs‐treated animals.

Finally, locomotor activity was assessed in two studies [22, 31] with UCMSCs treatment significantly increasing vertical activity (SMD = 1.76, 95% CI: 1.00 to 2.51, *I* ^2^ = 0%), vertical movement time (SMD = 2.09, 95% CI: 0.72 to 3.45, *I* ^2^ = 63%), and the number of vertical movements (SMD = 2.08, 95% CI: 0.68 to 3.48, *I* ^2^ = 65%) (Figure 5G–I).

### 3.7 UCMSCs Treatment Regulated the Levels of Inflammatory Cytokines and Promoted Microglial Polarization in IS Animals

**FIGURE 6:**
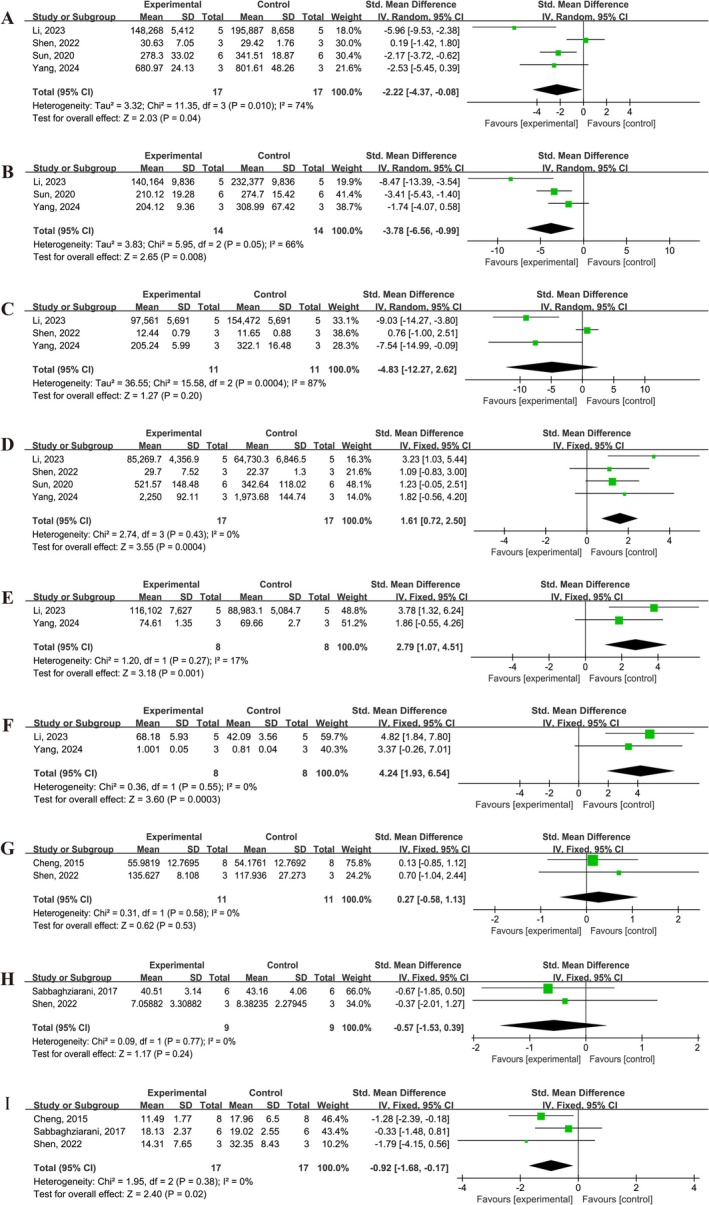
Forest plot showing the efficacy of UCMSCs on levels in brain tissue or serum of IS animals. (A) IL‐1β in brain. (B) IL‐6 in brain. (C) TNF‐α in brain. (D) IL‐10 in brain. (E) IL‐4 in brain. (F) TGF‐β in brain. (G) IL‐1β in serum. (H) IL‐6 in serum. (I) TNF‐α in serum.

The effects of UCMSCs treatment on inflammatory cytokines in brain tissue or serum of animals with IS was evaluated in several studies [13, 15, 27, 34, 36, 40]. The results indicated that UCMSCs treatment significantly reduced the levels of pro‐inflammatory cytokines, including IL‐1β (SMD = −2.22, 95% CI: −4.37 to −0.08, *I* ^2^ = 74%), IL‐6 (SMD = −2.22, 95% CI: −6.56 to −0.99, *I* ^2^ = 66%), and TNF‐α (SMD = −4.83, 95% CI: −12.27 to 2.62, *I* ^2^ = 87%) in brain tissue (Figure 6A–C). In contrast, UCMSCs treatment increased the levels of anti‐inflammatory cytokines, including IL‐10 (SMD = 1.61, 95% CI: 0.72 to 2.50, *I* ^2^ = 0%), IL‐4 (SMD = 2.79, 95% CI: 1.07 to 4.51, *I* ^2^ = 17%), and TGF‐β (SMD = 4.24, 95% CI: 1.93 to 6.54, *I* ^2^ = 0%) in brain tissue of IS animals (Figure 6D–F).

However, no significant differences were observed in the serum levels of IL‐1β (SMD = 0.27, 95% CI: −0.58 to 1.13, *I* ^2^ = 0%), and IL‐6 (SMD = −0.57, 95% CI: −1.53 to 0.39, *I* ^2^ = 0%) between the control and UCMSCs‐treated groups (Figure 6G,H). In contrast, UCMSCs treatment significantly reduced the serum level of TNF‐α (SMD = −0.92, 95% CI: −1.68 to −0.17, *I* ^2^ = 0%) (Figure 6I).

**FIGURE 7:**
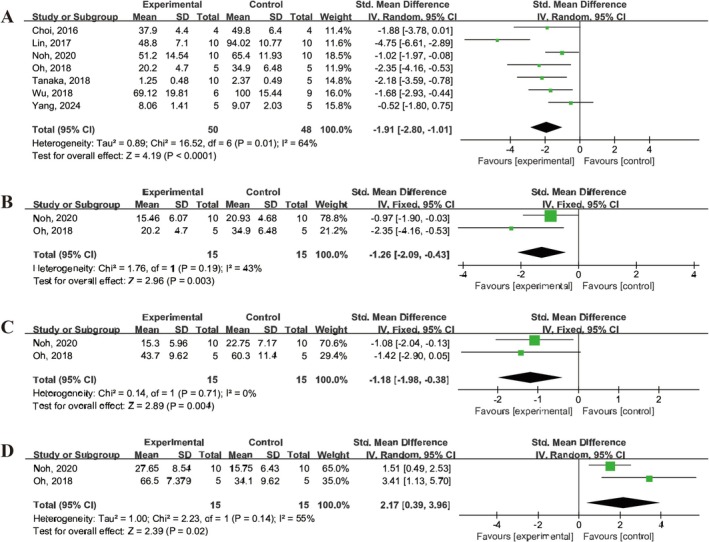
Forest plot demonstrating the efficacy of UCMSCs on microglial polarization in IS animals. (A) Iba1^+^ (%). (B) ED1^+^ (%). (C) Proportion of iNOS^+^/ED1^+^ cells. (D) Proportion of CD206^+^/ED1^+^ cells.

In terms of microglial polarization, seven studies [20, 29, 32, 33, 37, 39, 40] reported a decrease in the percentage of activated Iba‐1 positive cells in microglia following UCMSCs treatment (SMD = −1.91, 95% CI: −2.80 to −1.01, *I* ^2^ = 64%) (Figure 7A). Two studies [32, 33] reported that UCMSCs treatment reduced the proportion of pro‐inflammatory microglia in IS animals, as evidenced by a decreased number of ED1^+^ cells (SMD = −1.26, 95% CI: −2.09 to −0.43, *I* ^2^ = 43%) and the proportion of iNOS^+^/ED1^+^ cells (SMD =  −1.18, 95% CI: −1.98 to −0.38, *I* ^2^ = 0%) (Figure 7B,C). Furthermore, two studies [23, 27] reported an increase in the proportion of anti‐inflammatory microglia, as demonstrated by elevated the proportions of CD206^+^/ED1^+^ cells following UCMSCs treatment (SMD = 2.17, 95% CI: 0.39 to 3.96, *I* ^2^ = 55%) (Figure 7D).

### 3.8 UCMSCs Treatment Promoted Neuronal Proliferation and Inhibited Apoptosis in the Brain Tissue of IS Animals

**FIGURE 8:**
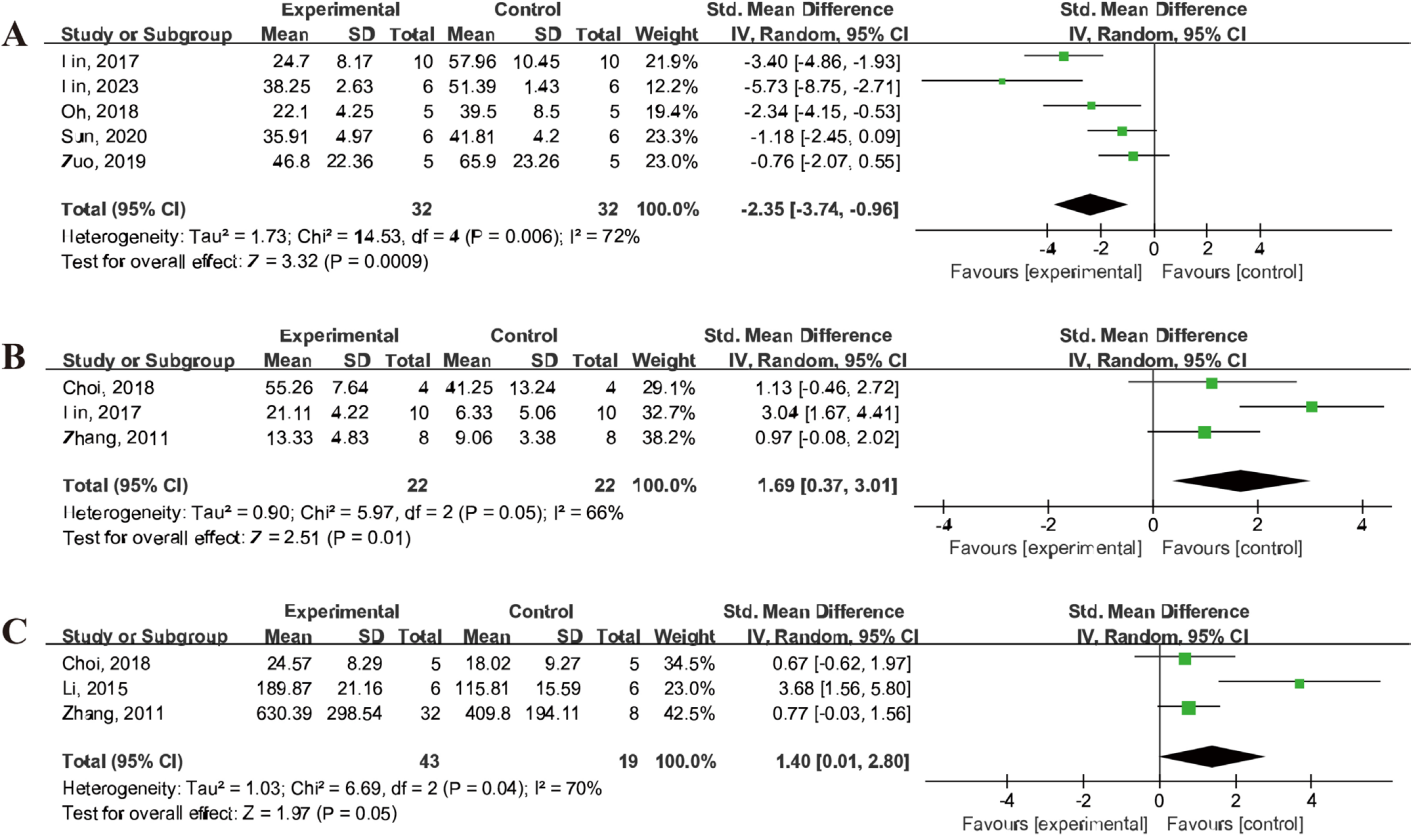
Forest plot showing the efficacy of UCMSCs on apoptosis and proliferation in IS animals. (A) Apoptosis rate. (B) Proliferation of neuroblasts. (C) BrdU‐labeled cells proliferating.

The apoptosis rate following IS was assessed in five studies [29, 30, 33, 36, 44], which indicated that treatment with UCMSCs significantly reduced the apoptosis rate in the brain tissue of IS animals (SMD = −2.35, 95% CI: −3.74 to −0.96, *I* ^2^ = 72%) (Figure 8A). Additionally, three studies [21, 29, 41] reported on neuronal proliferation in IS animals and demonstrated that UCMSCs treatment enhanced the proliferation of neuroblasts, as indicated by DCX staining (SMD = 1.69, 95% CI: 0.37 to 3.01, *I* ^2^ = 66%) (Figure 8B). Furthermore, three studies [21, 26, 41] reported an increase in the number of BrdU‐labeled proliferating cells in the brains of IS animals treated with UCMSCs (SMD = 1.40, 95% CI: 0.01 to 2.80, *I* ^2^ = 70%) (Figure 8C).

### 3.9 UCMSCs Treatment Promoted Angiogenesis in IS Animals

**FIGURE 9:**
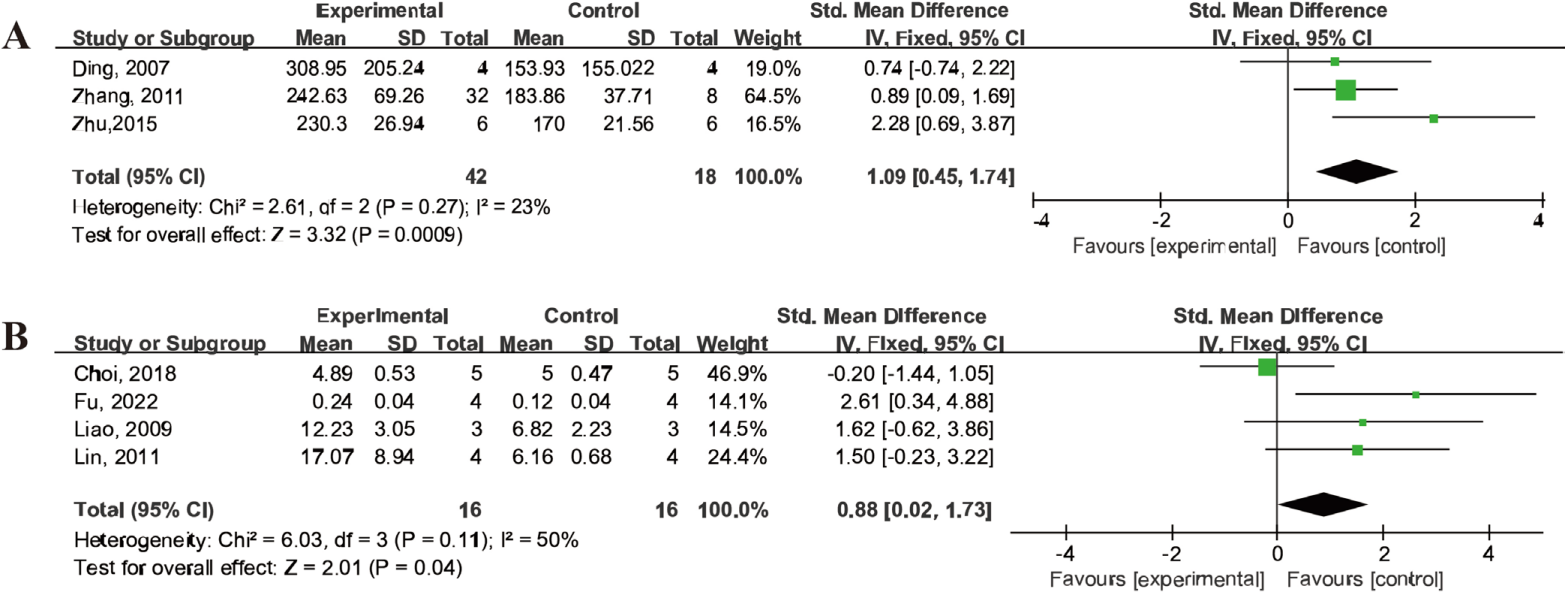
Forest plot illustrating the efficacy of UCMSCs on vessel density in IS animals. (A) Vessel density. (B) Percentage of vessel density.

Three studies [22, 41, 43] reported vessel density following IS in animals, while four studies [12, 14, 21, 28] demonstrated the percentage of vessel density. The results showed that UCMSCs treatment enhanced the ipsilateral vessel density (SMD = 1.09, 95% CI: 0.45 to 1.74, *I* ^2^ = 23%) and increased the percentage of vessel density (SMD = 0.88, 95% CI: 0.02 to 1.73, *I* ^2^ = 50%) (Figure 9A,B).

## 4 Discussion

To our knowledge, this meta‐analysis represents the first comprehensive review summarizing the efficacy of UCMSCs in treating IS in animal models and investigating their underlying mechanisms. A total of 30 articles were thoroughly examined, confirming the positive impact of UCMSCs on neurological function recovery in IS. The comprehensive results are summarized in Table 2.

**TABLE 2:** Summary table of meta‐analysis results.

Measures	Studies	Effect Estimate	Heterogeneity (*I* ^2^, *p*)
Percentage of infarction area	5	−1.26 [−1.88, −0.64]	*I*² = 64%, *p* < 0.0001
Percentage of infarction volume	8	−0.54 [−0.88, −0.20]	*I*² = 22%, *p* = 0.002
Infarct volume size	7	−1.88 [−2.62, −1.14]	*I*² = 63%, *p* < 0.00001
Brain water content	2	−1.50 [−3.06, 0.07]	*I*² = 73%, *p* = 0.06
mNSS	11	−1.03 [−1.45, −0.62]	*I*² = 61%, *p* < 0.00001
Zea Longa	2	−1.54 [−4.70, 1.63]	*I*² = 94%, *p* = 0.34
Rotarod test	5	0.96 [0.31, 1.60]	*I*² = 78%, *p* = 0.004
Retention time	3	2.71 [0.98, 4.45]	*I*² = 86%, *p* = 0.002
Adhesive removal test	3	−0.94 [−1.47, −0.42]	*I*² = 23%, *p* = 0.0004
Cylinder test	2	1.80 [0.74, 2.85]	*I*² = 70%, *p* = 0.0008
Morris water maze	3	−1.31 [−1.90, −0.73]	*I*² = 0%, *p* < 0.0001
Recovery of body asymmetry test	2	1.28 [0.59, 1.98]	*I*² = 0%, *p* = 0.0003
Vertical activity	2	1.76 [1.00, 2.51]	*I*² = 0%, *p* < 0.00001
Vertical movement time	2	2.09 [0.72, 3.45]	*I*² = 63%, *p* = 0.003
Number of vertical movements	2	2.08 [0.68, 3.48]	*I*² = 65%, *p* = 0.004
Brain_IL‐1β	4	−2.22 [−4.37, −0.08]	*I*² = 74%, *p* = 0.04
Brain_IL‐6	3	−3.78 [−6.56, −0.99]	*I*² = 66%, *p* = 0.008
Brain_TNF	3	−4.83 [−12.27, 2.62]	*I*² = 87%, *p* = 0.20
Brain_IL‐4	2	2.79 [1.07, 4.51]	*I*² = 17%, *p* = 0.001
Brain_IL‐10	4	1.61 [0.72, 2.50]	*I*² = 0%, *p* = 0.0004
Brain_TGF‐β	2	4.24 [1.93, 6.54]	*I*² = 0%, *p* = 0.0003
Serum_IL‐1β	2	0.27 [−0.58, 1.13]	*I*² = 0%, *p* = 0.53
Serum_IL‐6	2	−0.57 [−1.53, 0.39]	*I*² = 0%, *p* = 0.24
Serum_TNF	3	−0.92 [−1.68, −0.17]	*I*² = 0%, *p* = 0.02
Iba‐1(%)	7	−1.91 [−2.80, −1.01]	*I*² = 64%, *p* < 0.0001
ED1^+^ (%)	2	−1.26 [−2.09, −0.43]	*I*² = 43%, *p* = 0.003
iNOS^+^/ED1^+^	2	−1.18 [−1.98, −0.38]	*I*² = 0%, *p* = 0.004
CD206^+^/ED1^+^	2	2.17 [0.39, 3.96]	*I*² = 55%, *p* = 0.02
Apoptosis	5	−2.35 [−3.74, −0.96]	*I*² = 72%, *p* = 0.0009
DCX^+^	3	1.69 [0.37, 3.01]	*I*² = 66%, *p* = 0.01
BrdU‐labeled cells proliferating	3	1.40 [0.01, 2.80]	*I*² = 70%, *p* = 0.05
Vessel density	3	1.09 [0.45, 1.74]	*I*² = 23%, *p* = 0.0009
Percentage of vessel density	4	0.88 [0.02, 1.73]	*I*² = 50%, *p* = 0.04

In recent years, several meta‐analyses have been conducted on preclinical studies of MSCs for the treatment of IS [17, 45, 46, 47]. One study focused on adipose‐derived stem cells (ADSCs) [46], another analyzed bone marrow‐derived stem cells (BMSCs) [47], while most meta‐analyses covered all types of MSCs with a specific emphasis on BMSCs [17, 45]. Notably, none have performed a separate evaluation of UCMSCs in IS animals. These studies primarily evaluated various outcome measures such as infarct volume, neurologic scores, and behavioral tests, all demonstrating positive improvements [17, 45, 46, 47]. However, our study further assessed additional outcome indicators, including inflammatory cytokine levels, vascular density, and neuronal proliferation and apoptosis in IS animals. Our findings revealed that UCMSCs treatment exhibited positive effects in regulating inflammatory cytokines, promoting angiogenesis, inhibiting apoptosis, and promoting nerve regeneration. This comprehensive analysis provides a novel perspective on the multidimensional role of UCMSCs in treating IS.

A meta‐analysis conducted in 2020 demonstrated that MSCs derived from various sources enhance neurological recovery in IS animals, encompassing a total of 76 studies involving different types of MSCs, with seven studies specifically utilizing UCMSCs [17]. Notably, subgroup analysis revealed that animals receiving ADSCs or autologous BMSCs exhibited significantly superior performance in the rotarod test compared to those receiving allogeneic BMSCs or UCMSCs [17]. Conversely, animals treated with UCMSCs displayed the most favorable outcomes in the adhesive removal test [17]. Importantly, our study focused exclusively on UCMSCs administration, further supporting their significant positive impact on promoting behavioral recovery in IS animals. Furthermore, clinical investigations have indicated notable improvements in upper limb muscle strength, spasticity, and fine motor function among patients treated with UCMSCs therapy during a 12‐month follow‐up period [16].

Liao et al. have reported that UCMSCs homing to the ischemic boundary zone exhibit robust survival capabilities for a minimum of 5 weeks, suggesting potential long‐term neural repair and protective benefits [28]. Most included studies in this meta‐analysis utilized male animals to construct ischemic stroke models. This choice may be attributed to the neuroprotective effects of estrogen in female rodents, leading to significant sex differences in post‐stroke brain damage [48]. There may be sex‐based disparities in the efficacy of MSC therapy for IS, with male animals potentially being more responsive to treatment [48]. However, MSC therapy has been shown to improve neurological function in both male and female animals following stroke [48]. Further sex‐specific investigations are warranted to comprehensively assess the therapeutic potential of MSCs and to provide more precise guidance for clinicaluse.

The infarcted brain size and volume are the two principal outcomes used to evaluate the neural protective benefits of MSCs in ischemic stroke. Infarct size yielded a relatively similar effect, while infarct volume appears to have a smaller effect size in our meta‐analysis. This discrepancy may be attributed to variations in the timing of UCMSC transplantation, ways of administration, observation period, and the two‐dimensional versus three‐dimensional spatial calculations of the infarct region.

Despite the considerable therapeutic potential of UCMSCs, achieving optimal dosing remains a challenge in both clinical and preclinical investigations. Oh et al. found that intravenous transplantation of at least 1 × 10^6^ UCMSCs during the acute phase of cerebral ischemia could induce behavioral and histological improvements in a rat model of MCAO [33]. The dose–response study conducted by Zhang et al. showed that injection doses of UCMSCs at 3 × 10^6^ and 1 × 10^7^ resulted in significant improvements in neurological deficit scores and adhesive removal tests among IS animals, whereas doses at 3 × 10^5^ and 1 × 10^6^ did not yield improvement [41]. Shen et al. also demonstrated that early administration of relatively high doses of UCMSCs significantly enhanced neurological and motor function scores, as well as increased the rate of improvement in cerebral infarct size among IS animals, while lower doses had limited effects [15]. All this evidence suggests that UCMSCs are dose‐dependent, with moderate or high doses usually producing the best neural repair and protective benefits. Currently, no standardized dosage regimen exists, necessitating further research to determine the most effective dosage for optimizing the therapeutic efficacy of UCMSCs.

Additionally, the timing of UCMSCs transplantation has been found to influence its therapeutic efficacy. Among the included studies, earlier intervention resulted in better treatment outcomes, with the most significant effects observed when UCMSCs were administered within 24 h after stroke onset [33]. Most studies have demonstrated that intravenous transplantation of UCMSCs effectively enhanced neurological recovery in IS animals. However, Noh et al. reported no significant improvement in behavioral function or histological parameters after intravenous injection within the same 7‐week timeframe. This discrepancy may be attributed to the timing of UCMSCs infusion during the subacute stage of stroke.

The included studies mainly used IV and IC administration; each has its advantages and drawbacks. IC administration is a straightforward approach of delivering MSCs directly to the target site that can effectively promote brain tissue repair and regeneration [48]. However, it is an invasive method requiring a surgery that might develop complications such as bleeding, infection, and tissue damage. IV administration is considered a relatively secure procedure [49]. However, the homing potential of transplanted MSCs to target tissue after IV administration is inefficient, which might diminish therapeutic efficacy [37]. Although IV administration is a widely used non‐invasive technique in current clinical practice, the advantage of IC administration may be more apparent in clinical circumstances that require precision therapy.

In conclusion, further research is warranted to address these issues and determine the most optimal methods and procedures for clinical application, ensuring the full therapeutic potential of UCMSCs in treating nervous system injuries caused by IS.

## 5 Potential Mechanisms of Action of UCMSCs in the Treatment of IS

The potential mechanisms of action of UCMSCs in treating IS can be summarized as follows:

### 5.1 Immunity Regulation

Microglia and macrophages serve as resident immune cells in the central nervous system. Following a stroke, these cells can undergo phenotypic transformation into pro‐inflammatory M1 cells or anti‐inflammatory M2 cells [50]. M1 cells secrete pro‐inflammatory cytokines, such as IL‐1β, IL‐6, and TNF‐α, while M2 cells secrete anti‐inflammatory cytokines, such as IL‐10, IL‐4, and TGF‐β [51]. These cytokines jointly mediate the inflammatory injury of brain tissue.

This meta‐analysis demonstrated that UCMSCs have immunomodulatory properties, promoting microglia polarization, inhibiting the expression of pro‐inflammatory cytokines, and facilitating the release of anti‐inflammatory factors [38, 52, 53]. Additionally, Zhai et al. reported that UCMSC therapy upregulates mRNA expression levels of IL‐10 in the brain while downregulating the expression levels of TNF‐α [54]. This bidirectional regulation allows UCMSCs to play a protective role in the inflammatory environment after stroke.

### 5.2 Inhibition of Apoptosis and Promotion of Nerve Regeneration

Extensive neuronal apoptosis occurs in the peri‐infarct region following cerebral ischemia. This meta‐analysis revealed that UCMSCs treatment can inhibit apoptosis and promote neuronal proliferation through various mechanisms. Evidence indicates that UCMSCs treatment resists apoptosis, reduces neuronal loss, and promotes the regeneration of neuroblasts and proliferation of neuroblasts in regions such as the subventricular zone [32]. Additionally, UCMSCs treatment promotes the expression of chemokine CXCR4 and SDF‐α in brain tissue, thereby facilitating neural repair, proliferation, and maturation [15].

In the ischemic region following stroke, the expression of apoptosis‐related proteins has been shown to increase; however, UCMSCs treatment has been found to suppress the expression of pro‐apoptotic proteins, including Bax, Bcl‐2, and caspase‐3 [36]. Additionally, UCMSCs treatment has been shown to elevate the expression of neural stem cell markers Sox2 and Nestin [39].

### 5.3 Promotion of Angiogenesis

Angiogenesis is recognized as a crucial protective mechanism that promotes nerve regeneration and functional recovery after IS [55]. The findings of this meta‐analysis indicated that UCMSCs treatment can increase vessel density in the area of cerebral infarction in IS animals. Transplanted UCMSCs migrate to the ischemic border zone, induce angiogenesis, increase vascular density on the ischemic side, and help improve the blood supply and functional recovery of the brain tissue [22, 32].

Chouw et al. showed that UCMSCs secrete significantly higher levels of VEGF and HGF compared to MSCs derived from other sources, suggesting that UCMSCs may be more effective in promoting angiogenesis [49]. Additionally, the activation of Notch1 signaling following stroke further stimulates UCMSCs to secrete VEGF‐A, thereby enhancing angiogenesis [43].

### 5.4 Neurotrophic Effect

The neurotrophic factors brain‐derived neurotrophic factor (BDNF) and glial cell line‐derived neurotrophic factor (GDNF) play crucial roles in neuronal survival, differentiation, and synaptic plasticity. MSCs possess paracrine properties and have been shown to enhance the expression of various neurotrophic factors. Studies indicated that UCMSCs treatment leads to an elevation in BDNF and GDNF mRNA expression levels in animal brain tissue with IS, resulting in reduced cerebral infarction and improved learning and memory abilities [35, 56]. However, Wu et al. did not observe a significant improvement in BDNF mRNA expression following UCMSCs transplantation; instead, they found a significant increase in GDNF mRNA expression in the cortex of the transplanted side [39].

### 5.5 Suppression of Oxidative Stress

Oxidative stress is an important mechanism involved in the pathogenesis of IS, with elevated levels of oxidative stress markers observed in affected patients [57]. Due to the limited number of relevant studies, it was not possible to combine the outcome indicators; however, existing studies have shown that UCMSCs treatment can reduce the levels of ROS and lipid peroxide malondialdehyde, while increasing levels of the antioxidant superoxide dismutase and glutathione. This treatment has been associated with a reduction in oxidative stress damage in brain tissue after ischemia [23, 27, 44]. Additionally, the activation of the antioxidant transcription factor Nrf2 has been observed, further contributing to the protective effects against oxidative stress [27, 44].

In recent years, multiple optimization strategies have been investigated to improve the neural protective efficacy of UCMSCs, including combination pharmacotherapy, preconditioning, and genetic engineering. Taking an example, curcumin‐hUCMSCs combination therapy has proven to be more effective than single hUCMSCs therapy in protecting neurons from injury and reducing infarct volume, which could be related to the ability of curcumin to enhance the anti‐inflammatory and antioxidant properties of polarized microglia [27]. Besides this evidence, studies also reported that preconditioning UCMSCs with hypoxic conditions or inflammatory factors is capable of upregulating immune regulation‐related genes, thereby enhancing the immunosuppressive capabilities of MSCs [28, 58]. HO‐1 overexpressed UCMSCs increased the secretion of BDNF and anti‐inflammatory factors in ischemic regions, significantly mitigating secondary inflammatory injury in MCAO mice and decreasing mortality [40]. Furthermore, UCMSCs modified with PD‐L1 and AKT have been used in the treatment of ischemic stroke, which facilitates brain tissue recovery by modulating the immune microenvironment, attenuating immune responses, and reducing neuronal death [30]. In the future research of UCMSCs, researchers should focus on optimizing protocols and integrating these advancements with existing clinical therapies.

## 6 Limitations

This study has several limitations that should be considered. First, only three commonly used English databases were searched, and only studies with full texts in English were included. Chinese databases and literature in other languages were not extracted, potentially leading to some language bias. Additionally, the small number of studies included in some analyses may raise concerns regarding the reliability of the combined findings. Moreover, studies utilizing various doses, treatment windows, or transplantation routes for UCMSCs may have contributed to a lower observed therapeutic efficacy when data were aggregated for comprehensive analysis.

## 7 Conclusion

In conclusion, this meta‐analysis demonstrates that UCMSCs treatment has a positive effect on promoting neurological function recovery after IS. These effects may be attributed to the regulation of immune responses, inhibition of cell apoptosis, and promotion of angiogenesis. This study provides valuable guidance for both basic research and clinical applications involving UCMSCs in the treatment of IS. Future efforts should focus on conducting more high‐quality preclinical and clinical studies, as well as accelerating the translation of clinical applications.

## Author Contributions

All authors contributed to the article and agreed to submit the manuscript. Shenghang Zhang and Jianhong Li supervised the project and provided suggestions during the entire research process. Renli Wei and Minguang Yang contributed to literature screening, data processing and analysis, and wrote the manuscript. Yue Cao, Shuqian Qiu, Xu Fan, Muxuan Fang, Chen Li, and Cheng Shaojie participated in data extraction.

## Conflicts of Interest

The authors declare no conflicts of interest.

## Data Availability

The data that support the findings of this study are available from the corresponding author upon reasonable request.
